# A Pyrosequencing Assay for the Quantitative Methylation Analysis of *GALR1* in Endometrial Samples: Preliminary Results

**DOI:** 10.1155/2015/756359

**Published:** 2015-10-04

**Authors:** Christine Kottaridi, Nikolaos Koureas, Niki Margari, Emmanouil Terzakis, Evripidis Bilirakis, Asimakis Pappas, Charalampos Chrelias, Aris Spathis, Evangelia Aga, Abraham Pouliakis, Ioannis Panayiotides, Petros Karakitsos

**Affiliations:** ^1^Department of Cytopathology, “ATTIKON” University Hospital, University of Athens Medical School, 1 Rimini, Haidari, 12462 Athens, Greece; ^2^2nd Department of Gynecology, St. Savas Cancer Hospital, 171 Alexandras Avenue, 11522 Athens, Greece; ^3^Department of Obstetrics and Gynecology, IASO Hospital, 37-39 Kifisias Avenue, Maroussi, 15123 Athens, Greece; ^4^3rd Department of Obstetrics and Gynecology, “ATTIKON” University Hospital, 1 Rimini, Haidari, 12462 Athens, Greece; ^5^2nd Department of Pathology, “ATTIKON” University Hospital, University of Athens Medical School, 1 Rimini, Haidari, 12462 Athens, Greece

## Abstract

Endometrial cancer is the most common malignancy of the female genital tract while aberrant DNA methylation seems to play a critical role in endometrial carcinogenesis. Galanin's expression has been involved in many cancers. We developed a new pyrosequencing assay that quantifies DNA methylation of galanin's receptor-1 (*GALR1*). In this study, the preliminary results indicate that pyrosequencing methylation analysis of *GALR1* promoter can be a useful ancillary marker to cytology as the histological status can successfully predict. This marker has the potential to lead towards better management of women with endometrial lesions and eventually reduce unnecessary interventions. In addition it can provide early warning for women with negative cytological result.

## 1. Introduction

Endometrial cancer is the most common malignancy of the female genital tract. 80% of endometrial cancers occur in postmenopausal women with a mean of age 61 years at diagnosis [1]. According to already published data, the background incidence of endometrial cancer ranges from 0.6 to 6/1000 [2, 3]; thus endometrial cancer may potentially be a serious problem of public health, especially for postmenopausal women. Additionally, the duration of endometrial intraepithelial carcinoma can be of 8–12 years and of occult disease more than 5 years, thus providing plenty of time for detection in early stage. Endometrium is an easy to access organ, providing adequate and representative cytological material for examinations. Thus this material plays an important role for endometrial cancer detection and prevention.

One of the most important cancer hallmarks is aberrant DNA methylation [4]. However, it remains unclear when these changes take place and what is their precise role in the development of cancer. DNA methylation occurs when a methyl group is transferred to the 5′ position of a cytosine nucleotide adjacent to guanine (CpG). Usually CpGs are clustered in CpG islands and those that reside at gene's promoter region are normally unmethylated allowing the active transcription of the gene. In cancer cells a transcriptional silencing is observed due to methylation that is targeting the promoters of genes [5–7]. As far as endometrial carcinogenesis is concerned, there is accumulating evidence that except from the environment [8, 9] aberrant DNA methylation plays a critical role [10–12].

Galanin is a neuropeptide which belongs in a family of peptides whose expression has been involved in many cancers [13]. It regulates many biological and pathological functions through three different receptor subtypes (*GALR1*,* GALR2*, and* GALR3*) [14]. Loss of* GALR1* expression is associated with its promoter hypermethylation supporting the hypothesis that* GALR1* acts as a tumor suppressor gene [14–16].

In the present study, a new pyrosequencing approach was developed. The purpose was to assess this method and evaluate its utility to identify possible differences of DNA methylation status in* GALR1* promoter genomic region between normal and malignant endometrial samples.

## 2. Materials and Methods

### 2.1. Samples and DNA Extraction

*GALR1* DNA methylation was investigated in 61 specimens, coming from the sample that has been collected with the EndoGyn device. Samples were collected from women who were admitted to the 3rd Department of Obstetrics and Gynecology “ATTIKON” University Hospital, “Saint Savvas” Anticancer Hospital, Athens, Greece. All patients signed an informed consent form, while the study was approved by the bioethics committee of the hospitals.

The samples' analysis was performed at cytopathology Department and the histological evaluation at the 2nd department of pathology, “ATTIKON” University Hospital. The histological material was from dilation and curettage and/or hysterectomy. The mean age of women was 61.2 years ± 12.7 (minimum 33, maximum 86). For the histologically benign cases the mean age was 54.7 years ± 7.0 (minimum 47, maximum 72) and for the histologically malignant cases the mean age was 64.0 years ± 13.6 (minimum 33, maximum 86). The pooled *t*-test was used for the comparison of the histologically benign and malignant group ages; it gave *t* = −2.74, *p* = 0.0080 < 0.05, meaning that the two groups have statistically significant different ages. The studied cases were selected as follows: from the files of the cytopathology laboratory we extracted all cases that had histological correlation and available biological material for further analysis. From these cases we randomly selected 61.

The cytological material was collected by gynecologists using the EndoGyn device especially designed for endometrial sampling. After the collection of endometrial material, the EndoGyn device was withdrawn and immersed into a vial containing 30 mL of appropriate hemolytic, mucolytic, and proteinolytic solution (CytoLyt, Cytyc Corporation), removing the unwanted background which limits cytological diagnosis [17]. LBC ThinPrep methodology was performed as described in detail in our previous work [18, 19]. In the cytopathology laboratory liquid-based cytology is applied as routine because it allows standardized and reproducible endometrial preparations and additional material remains for ancillary tests. For each case we prepared one slide stained with the Papanicolaou technique using an automated staining machine (Varistain; Thermo Electron Corporation [formerly Shandon], Runcorn, UK) and the remaining material was used for ancillary techniques including quantification of DNA methylation in* GALR1*.

The routine diagnostic procedure of the lab for reporting endometrial cytology was conformant to the 1994 World Health Organization (WHO) classification scheme [19, 20]. Thus, in this study the cytological diagnosis was provided as follows: benign, polyp, hyperplasia without cytological atypia (subsequently referred as hyperplasia WoA), atypical, and malignant. Cases diagnosed as atypical were subject to differential diagnosis between hyperplasia with atypia and well differentiated adenocarcinoma. In terms of increasing severity, the cytological categories in this study formed four groups: benign, hyperplasias WoA and polyp (subsequently referred to as HWoA-P) atypical, and malignant.

The histological material included cases diagnosed as benign, polyp, hyperplasia without atypia (hyperplasias WoA), and malignant, the last category includes endometrioid, mucinous, clear cell carcinoma (subsequently referred as CCC), serous carcinomas, and mixed and carcinosarcoma.

Concerning DNA methylation tests, DNA was extracted from 1.5 mL preserved cells using the PureLink Genomic DNA Mini Kit (Invitrogen, USA) according to manufacturer instructions. At the end of DNA extraction method, 100 *μ*L of eluted DNA was recovered and stored at −20°C.

### 2.2. Sodium Bisulfate Treatment and Pyrosequencing

All DNAs were bisulfite converted using the EpiTect Bisulfite Kit (Qiagen, Hilden, Germany), according the manufacturer's instructions. All bisulfite converted samples were checked with *β*-actin (ACTB) and their concentration was adjusted to be the same as control's (10 ng/*μ*L). The housekeeping gene ACTB was chosen as an internal reference [21]. Primers were designed using PyroMark Assay design SW 2.0 (Qiagen, Hilden, Germany). Assays were designed to target a region within the CpG island located around the transcription start site of the gene coding for* GALR1*. The selection of this region was based on Doufekas et al. [15] where it is shown that there is an increase of mean methylation in cancerous endometrium when it is compared with normal. PCRs were performed using the following primers: sense-GALFd GTTTAGGGGGAAGTTTAGATTT, antisense-GALRd BTN- ACCCCCAACTCCATAACCC, and sequencing forward-sGALd GGGGGAAGTTTAGATTTT. All PCRs were performed with the PyroMark PCR kit (Qiagen, Hilden, Germany). The conditions for PCR amplifications were as follows: A 15-minute incubation at 95°C was followed by 45 cycles of 30 seconds at 94°C, 30 seconds at 56°C, and 30 seconds at 72°C. A ten-minute elongation step at 72°C completed the PCR amplification.

A total of thirteen CpGs were analyzed. The pyrosequencing reactions were conducted using PyroMark Q24 Advanced CpG reagents and a PyroMark Q24 Instrument upgraded with the PyroMark Q24 Advanced software (Qiagen, Hilden, Germany). The pyrograms were analyzed using the CpG mode of the PyroMark Q24 Advanced software, to determine the methylation percentage of each site as well as the overall mean methylation. Every PCR and methylation run included H_2_O, an EpiTect methylated and bisulfite converted, and an EpiTect unmethylated and bisulfite converted control human DNA (Qiagen, Hilden, Germany).

### 2.3. Statistical Analysis

The statistical analysis was performed by SAS 9.3 for Windows (SAS Institute Inc., NC, USA) [22, 23]. Within the analysis we have included an additional variable, namely, the mean methylation level calculated as the total of methylation levels for all positions divided by the number of positions that methylation measurement was successful (see Supplementary Information in Supplementary Material available online at http://dx.doi.org/10.1155/2015/756359 for the variables involved in the study).

The first step of the analysis was to examine the correlation of methylation percentages for each individual position; this analysis was performed by calculating the Pearson correlation coefficients for all possible pairs.

The second step was to examine if the mean methylation level is different for the cytological category groups and for this reason we produced boxplots of the mean methylation and performed regression analysis in order identify if there is a relation governing the mean methylation percentage and the disease severity as this is expressed in the cytological result.

The next step of the analysis was to extract the receiver operating characteristics curves (ROC) for each methylation position and identify if there are individual methylation positions that can be used as discriminators for the detection of endometrial malignancies. We used as cut-off level the histological categories benign and malignant.

Finally we tried to identify a cut-off value of the mean methylation level in order to characterize a sample as histologically benign or malignant. For this purpose the data were separated into two sets: the training set used to identify the threshold and the test set used to test the performance of the method on unknown data. Comparison with the cytological approach was performed as well. The algorithms for the determination of the optimum threshold values were calculated with in-house developed software for the MATLAB environment (The MathWorks, Inc., Natick, Massachusetts, USA).

Within our measurements, there were positions that were assigned as unsuccessful by the PyroMark Advanced software. Specifically from the 61 cases it was possible to measure methylation in all 13 positions in 43 cases (70.49%), in 14 cases (22.95%) in 12 positions, and in 4 cases (6.56%) in 12 positions.

## 3. Results

We developed a pyrosequencing assay that recognizes part of the CpG island near the transcription start site of* GALR1*. To design a set of primers we used as target sequence a region that was previously shown as being highly methylated in endometrial cancer using a MethyLight assay [15]. The reproducibility of the assay was checked when different runs were performed by testing two samples characterized as unmethylated and two samples characterized as methylated when compared with the negative control, in previous runs. The level of the methylation that was observed was almost similar. All raw pyrograms were evaluated. The quantitative methylation levels of the CpG sites in the region sequenced were analyzed by the Advanced PyroMark software.

The correlation of the cytological with the histological diagnosis is presented in Table 1. Cytologically 12 (19.67%) cases were negative: one case was secretory endometrium, one case was proliferative, and the remaining ten cases atrophic endometrium. One case was characterized cytologically as polyp (1.64%) and five cases (8.20%) were characterized as hyperplasias without atypia, four cases (8.20%) were found cytologically as atypical, and 39 (63.93) as malignant. From the 39 cytologically malignant cases 36 were diagnosed in cytology as adenocarcinomas, one case as squamous cell carcinoma, and two cases as malignant unless otherwise specified. In our material there were no cases of hyperplasia with atypia.

**Figure 1:**
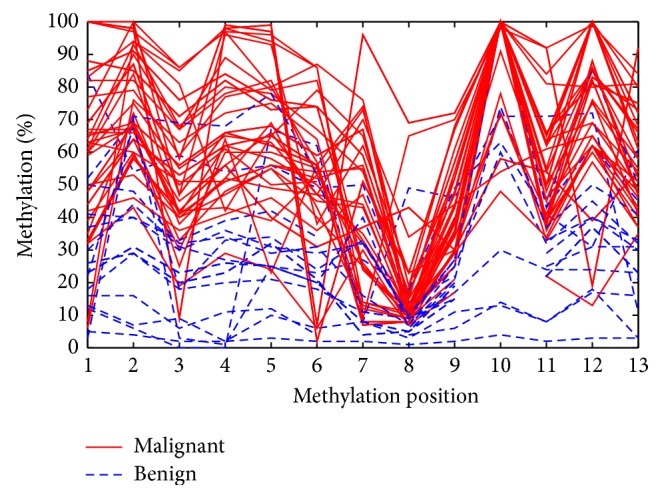
Methylation percentages for each methylation position for the studied samples.

A plot indicating methylation percentages for each CpG position on the *x*-axis and methylation percentage on the *y*-axis for all involved samples is provided in Figure 1. The red solid lines representing the malignant cases are mostly concentrated on the upper part in contrast to the benign cases. This figure depicts as well that methylation is highly correlated in various positions; the correlation analysis via the Pearson correlation coefficient is presented in the supplement.

**Figure 2:**
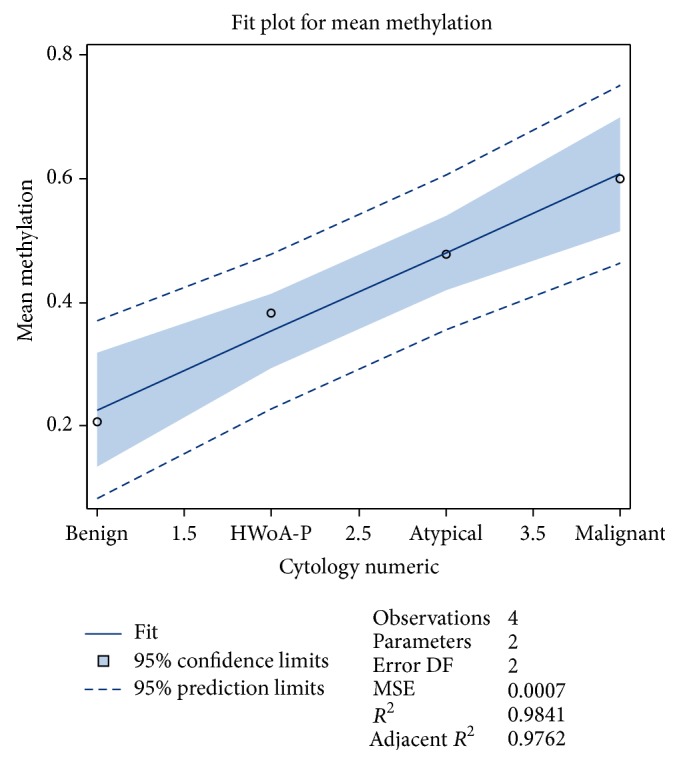
Fit plot for mean methylation in relation to the severity of the cytological diagnosis.

In relation to the lesion severity as this is depicted by the cytological result (expressed in numeric form; see Supplement, CytologyNumeric variable) we performed regression analysis (see Figure 2) and this produced a linear relation between the mean methylation and the cytological outcome: mean  methylation = 0.10 + 0.13*∗*CytologyNumeric. The line fit is almost perfect as *R* ^2^ = 98.41%. The positive slope (0.13) indicates that from one cytological category to the other the mean methylation percentage increases by 13%.

**Figure 3:**
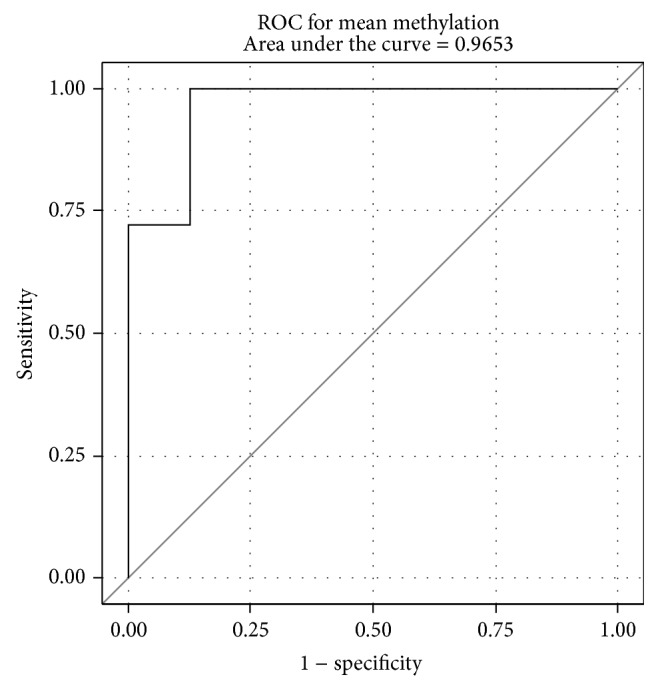
ROC curves of the mean methylation in relation to the histological outcome using as categories benign and malignant.

Subsequently we calculated the ROC curve for mean methylation, in order to evaluate the potential value of the mean methylation percentage as a predictor of the status of malignancy as this is defined by the histological golden standard. The ROC curve appears in Figure 3. A more detailed ROC analysis for all methylation positions appears in the Supplement.

As the mean methylation has the best performance, in terms of ROC curve analysis, the next step was to identify a threshold and separate the histologically benign from histologically malignant cases. About 50% (9 histologically benign and 22 malignant cases) were randomly selected and used to identify the optimum threshold. This was determined using a procedure already described in the bibliography [24]; specifically a broad range of thresholds was used starting from 20% and increasing up to 100% with an increment step of 1%; for each threshold value we calculated the percentage of the cases that were correctly classified (overall accuracy). As most suitable threshold, the threshold that maximized the overall accuracy on the training set was selected. The optimal threshold using this approach was 37%. Thus an algorithm was able to be produced: “if the mean methylation level is higher than 37% then the sample is considered as histologically malignant and otherwise as benign.”

Using this threshold from the 9 benign cases of the training set, 8 were classified as benign while all malignant cases (22) were classified correctly; this results in an overall accuracy of 96.77%, sensitivity of 100.00%, and specificity of 88.89%. By applying the same threshold on the test set (9 histologically benign and 21 malignant cases) it was possible to classify correctly 8 out of the 9 benign cases and 20 out of the 21 malignant cases. Overall accuracy = 93.33%, sensitivity = 95.24%, and specificity = 88.89%. The comparison of the overall accuracy percentages via the *χ*-square test proved that the two values are not statistically different (*χ* ^2^ = 0.00084, *p* = 0.98 > 0.05) and thus the proposed method was stable on unknown data (despite the small sample number).

A figure depicting the overall accuracy for the training set, test set, and the complete data set for the various threshold values appears in the Supplement.

In this study all cytological cases were by design in agreement with the histological result (see Table 1); thus the overall accuracy of the cytological examination was by design 100%; however the comparison of the overall accuracy of the proposed method on the complete data set (overall accuracy = 95.08, sensitivity = 97.67, and specificity = 88.89%) via the *χ*-square test proved that there was no statistically significant difference (*χ* ^2^ = 1.368, *p* = 0.24 > 0.05). Thus statistically the proposed mean methylation thresholding method may be considered equivalent to cytology.

## 4. Discussion

Epigenetics are changes in gene expression which are not a result of altered nucleotide sequence [25]. As it was previously described, the definition of methylation levels could identify patients with different clinical characteristics [26]. Having the advantage of calculating the mean methylation of each CpG during pyrosequencing and expressing it as an absolute value, this method is featured as gold standard technology for quantitative methylation studies.

The neuropeptide galanin elicits a range of biological effects by interaction with specific G-protein-coupled receptors. Galanin receptors are seven-transmembrane proteins shown to activate a variety of intracellular second-messenger pathways.* GALR1* inhibits adenylyl cyclase via a G protein of the Gi/Go family [27]. According to Rauch and Kofler [13], the galanin peptide expression is studied in pheochromocytoma, pituitary adenoma, neuroblastic tumors, gastrointestinal cancer, squamous cell carcinoma, brain tumors, melanoma, breast cancer, and embryonal carcinoma. In another study it is highlighted that the galanin promoter methylation profile could be an important marker predicting the clinical outcome of head and neck squamous cell carcinoma [16].

As far as the endometrium is concerned, the biological function of the galanin system is not studied extensively. According to Doufekas et al. [15], there is an evidence that* GALR1* suppression possibly plays a role in endometrial cancer development. The authors found that* GALR1* methylation is among the most frequent and consistent methylation differences between benign and cancerous endometrium.

In our study we found that the methylation percentage was increased from the benign lesions to the HWoA-P as well as to atypical and malignant lesions (see Figure 2 related to the mean methylation), a fact that indicates that methylation of* GALR1* may play a role in carcinogenesis. This finding led to subsequent analysis using the histological outcome as gold standard; the high percentages of the areas under the ROC for almost all measured positions (see Supplement) and especially for the mean methylation level proved that this test is not only reliable but accurate as well. A strong correlation of the methylation percentages in numerous positions was observed during the analysis of the results of the present study. This could be an indication for retaining accuracy in the overall mean methylation percentage; even if for laboratory reagents consuming or for time saving purposes, this method has to analyze less positions in a massive application.

A recent study describes that DNA hypermethylation in endometrial tissues quantified by pyrosequencing can also be identified in vaginal pool DNA collected via intravaginal tampon, combining thus a minimally invasive collection method with a high-throughput methodology for the early detection of endometrial cancer [28]. Although our present work discusses a possible role of the described pyrosequencing technology in the future as an indicator of malignancy, additional studies with larger series may in the future be substantiated and provide more evidence and confirm these preliminary results. It is important that methylation can be measured on cytological material by applying a sampling method that is well tolerated, easy to use, less painful than biopsy, and cost-effective. It additionally provides a generous and adequate material as long as endometrium is an easy to access organ for sampling which is representative of the lesion for cytological evaluation and the application of ancillary techniques [19].

The detailed analysis of the mean methylation for the two major histologically malignant subgroups (see Table 1), specifically for endometrioid carcinomas (34 cases, mean methylation = 59.99%, min = 39.56%, max = 82.86%, S.D. = 12.24%) versus nonendometrioid carcinomas (9 cases, mean methylation = 52.24%, min = 23.06%, max = 70.48%, S.D. = 14.13%), revealed that the mean methylation levels of the two groups do not differ significantly (*χ* ^2^ = 0.048, *p* = 0.94 > 0.05). Thus in the small data set the conclusion is that the mean methylation measurements do have potential to discriminate endometrioid from nonendometrioid carcinomas.

Similar analysis in the histologically benign subgroups which is negative (10 cases, mean methylation = 19.70%, min = 2.36%, max = 36.16%, S.D. = 10.77%) versus the group including polyp or hyperplasias without atypia (8 cases, mean methylation = 35.15%, min = 20.13%, max = 51.32%, S.D. = 10.62%) revealed that the mean methylation levels of these two groups are not different (*χ* ^2^ = 0.040, *p* = 0.84 > 0.05). Therefore the mean methylation level of a case seems not to have the potential to discriminate cases that may harbor a polyp or hyperplasia without atypia from healthy subjects.

According to the results, it is possible to define a reliable cut-off level (37%) in the mean methylation in order to discriminate histologically benign from malignant lesions. As the comparison of the cut-off method proved that there is no statistically significant difference compared to cytology alone, the pyrosequencing methylation analysis of* GALR1* promoter has the potential to be used in the future as an additional marker (or even single test) to the cytological examination, for better management of women with endometrial lesions, either towards a follow-up or for referral to dilatation and curettage. Eventually it could reduce unnecessary interventions and provide an early warning for women with negative cytology or borderline lesions with high methylation percentages.

## Supplementary Material

In the supplement the variables that were studied are collectively presented. The correlation analysis via the Pearson correlation coefficient revealed a positive correlation between the methylation levels in two positions. A ROC analysis was performed for all positions in order to examine the discriminative power of each individual methylation position.

## Figures and Tables

**Table 1:** Cytology/Histology correlation.

		Histology								
		Benign	Polyp	Hyperplasia WoA	Endometrioid	Carcinosarcoma	CCC	Serous	Mixed	Grand total
Cytology	Benign	10	2							12
	Polyp		1							1
	Hyperplasia WoA			5						5
	Atypical				2			1	1	4
	Malignant				32	1	2		4	39
	Grand total	10	3	5	34	1	2	1	5	61
