# Supplementary material for: A Pyrosequencing Assay for the Quantitative Methylation Analysis of GALR1 in Endometrial Samples: Preliminary Results
Source: Biomed Res Int. 2015 Oct 4;2015:756359. doi: 10.1155/2015/756359 (PMC4609388; doi:10.1155/2015/756359)
Supplement: Supplementary file 1 — In the supplement the variables that were studied are collectively presented. The correlation analysis via the Pearson correlation coefficient revealed a positive correlation between the methylation levels in two positions. A ROC analysis was performed for all positions in order to examine the discriminative power of each individual methylation position. [file 756359.f1.docx]

**Supplementary Information**

**Variables involved in the study**

The variables that were studied are collectively presented in table s1, along with their naming, especially for the cytological and cytological categorization there were created two variables (CytologyNumeric and Histology Numeric) where the severity of the lesion was coded by a number, a high number value indicates more severe lesions.

| **Variable Name** | **Description** |
| --- | --- |
| Sample ID | Sample Number, used to uniquely identify the case under study |
| Pos_01_MethPercentage | Percentage of Methylation at position 1 |
| Pos_02_MethPercentage | Percentage of Methylation at position 2 |
| Pos_03_MethPercentage | Percentage of Methylation at position 3 |
| Pos_04_MethPercentage | Percentage of Methylation at position 4 |
| Pos_05_MethPercentage | Percentage of Methylation at position 5 |
| Pos_06_MethPercentage | Percentage of Methylation at position 6 |
| Pos_07_MethPercentage | Percentage of Methylation at position 7 |
| Pos_08_MethPercentage | Percentage of Methylation at position 8 |
| Pos_09_MethPercentage | Percentage of Methylation at position 9 |
| Pos_10_MethPercentage | Percentage of Methylation at position 10 |
| Pos_11_MethPercentage | Percentage of Methylation at position 11 |
| Pos_12_MethPercentage | Percentage of Methylation at position 12 |
| Pos_13_MethPercentage | Percentage of Methylation at position 13 |
| Number of included methylations | Number of methylation tests successfully completed |
| MeanMethylation | Mean value of methylation for all positions |
| Age | Woman age |
| CytologyNumeric | Cytological result expressed as number: 1=Benign, 2=Hyperplasia Without Atypia and Polyp (HWoA-P), 3= Atypical , 4=Malignant |
| HistologyNumeric | Histological result expressed as number: 1=Benign, 2=Polyp, 3 Hyperplasia Without Atypia 4=Endometrioid, 5= Carcinosarcoma, 6= CCC, 7= Serous, 8=Mixed |

Table s1: Analyzed variables

**Correlation between methylation positions**

The correlation analysis via the Pearson correlation coefficient revealed that there is always a positive correlation between the methylation levels in two positions. In many pairs the correlation coefficient is very high: position 2 and 4 r=0.90223, positions 2 and 5 r=0.84649, positions 2 and 10 r= 0.83083, positions 2 and 13 r=0.94627, positions 2 and mean methylation r=0.93644, positions 4 and 5 r=0.89608, positions 4 and 13 r=0.88437 and position 4 and mean methylation r= 0.91860, position 5 with 11 r=0.82702, 5 with 13 r=0.82305 and 5 with mean methylation r=0.90201. 9 with 11 r=0.85697, 9 with 13 r=0.82658, 9 with mean methylation r=0.83981, 10 with mean methylation r= 0.87875, position 11 with 13 r=0.85807, 11 with mean methylation r=0.87914, in all cases it was p<.0001. Additionally there were identified numerous other methylation positions that the methylation levels were correlated more than 65%, p<0.0001. In contrast no single pair with negative correlation was found. This finding indicates that methylation percentage increases simultaneously in numerous positions.

The interested reader may consult figure s1 depicting the detailed correlation matrix between all positions (yellow color indicates high correlation coefficients).

| **Pearson Correlation Coefficients  Prob > \|r\| under H0: Rho=0  Number of Observations** | | | | | | | | | | | | | | |
| --- | --- | --- | --- | --- | --- | --- | --- | --- | --- | --- | --- | --- | --- | --- |
|  | **Pos_01_MethPercentage** | **Pos_02_MethPercentage** | **Pos_03_MethPercentage** | **Pos_04_MethPercentage** | **Pos_05_MethPercentage** | **Pos_06_MethPercentage** | **Pos_07_MethPercentage** | **Pos_08_MethPercentage** | **Pos_09_MethPercentage** | **Pos_10_MethPercentage** | **Pos_11_MethPercentage** | **Pos_12_MethPercentage** | **Pos_13_MethPercentage** | **MeanMethylation** |
| \| **Pos_01_MethPercentage** \| \| --- \| \|  \| | \| 1.00000 \| \| --- \| \|  \| \| 61 \| | \| 0.76430 \| \| --- \| \| <.0001 \| \| 61 \| | \| 0.50453 \| \| --- \| \| <.0001 \| \| 61 \| | \| 0.72814 \| \| --- \| \| <.0001 \| \| 61 \| | \| 0.67394 \| \| --- \| \| <.0001 \| \| 61 \| | \| 0.27845 \| \| --- \| \| 0.0298 \| \| 61 \| | \| 0.61504 \| \| --- \| \| <.0001 \| \| 58 \| | \| 0.21344 \| \| --- \| \| 0.0986 \| \| 61 \| | \| 0.63784 \| \| --- \| \| <.0001 \| \| 61 \| | \| 0.59327 \| \| --- \| \| <.0001 \| \| 47 \| | \| 0.63284 \| \| --- \| \| <.0001 \| \| 60 \| | \| 0.49032 \| \| --- \| \| <.0001 \| \| 58 \| | \| 0.75710 \| \| --- \| \| <.0001 \| \| 61 \| | \| 0.77951 \| \| --- \| \| <.0001 \| \| 61 \| |
| \| **Pos_02_MethPercentage** \| \| --- \| \|  \| | \| 0.76430 \| \| --- \| \| <.0001 \| \| 61 \| | \| 1.00000 \| \| --- \| \|  \| \| 61 \| | \| 0.71577 \| \| --- \| \| <.0001 \| \| 61 \| | \| 0.90223 \| \| --- \| \| <.0001 \| \| 61 \| | \| 0.84649 \| \| --- \| \| <.0001 \| \| 61 \| | \| 0.48239 \| \| --- \| \| <.0001 \| \| 61 \| | \| 0.55052 \| \| --- \| \| <.0001 \| \| 58 \| | \| 0.33512 \| \| --- \| \| 0.0083 \| \| 61 \| | \| 0.75411 \| \| --- \| \| <.0001 \| \| 61 \| | \| 0.83083 \| \| --- \| \| <.0001 \| \| 47 \| | \| 0.78069 \| \| --- \| \| <.0001 \| \| 60 \| | \| 0.67975 \| \| --- \| \| <.0001 \| \| 58 \| | \| 0.94627 \| \| --- \| \| <.0001 \| \| 61 \| | \| 0.93644 \| \| --- \| \| <.0001 \| \| 61 \| |
| \| **Pos_03_MethPercentage** \| \| --- \| \|  \| | \| 0.50453 \| \| --- \| \| <.0001 \| \| 61 \| | \| 0.71577 \| \| --- \| \| <.0001 \| \| 61 \| | \| 1.00000 \| \| --- \| \|  \| \| 61 \| | \| 0.74058 \| \| --- \| \| <.0001 \| \| 61 \| | \| 0.67904 \| \| --- \| \| <.0001 \| \| 61 \| | \| 0.74794 \| \| --- \| \| <.0001 \| \| 61 \| | \| 0.13224 \| \| --- \| \| 0.3224 \| \| 58 \| | \| -0.01410 \| \| --- \| \| 0.9141 \| \| 61 \| | \| 0.48254 \| \| --- \| \| <.0001 \| \| 61 \| | \| 0.66416 \| \| --- \| \| <.0001 \| \| 47 \| | \| 0.50361 \| \| --- \| \| <.0001 \| \| 60 \| | \| 0.86915 \| \| --- \| \| <.0001 \| \| 58 \| | \| 0.61381 \| \| --- \| \| <.0001 \| \| 61 \| | \| 0.76983 \| \| --- \| \| <.0001 \| \| 61 \| |
| \| **Pos_04_MethPercentage** \| \| --- \| \|  \| | \| 0.72814 \| \| --- \| \| <.0001 \| \| 61 \| | \| 0.90223 \| \| --- \| \| <.0001 \| \| 61 \| | \| 0.74058 \| \| --- \| \| <.0001 \| \| 61 \| | \| 1.00000 \| \| --- \| \|  \| \| 61 \| | \| 0.89608 \| \| --- \| \| <.0001 \| \| 61 \| | \| 0.43947 \| \| --- \| \| 0.0004 \| \| 61 \| | \| 0.45853 \| \| --- \| \| 0.0003 \| \| 58 \| | \| 0.38365 \| \| --- \| \| 0.0023 \| \| 61 \| | \| 0.71160 \| \| --- \| \| <.0001 \| \| 61 \| | \| 0.76610 \| \| --- \| \| <.0001 \| \| 47 \| | \| 0.78166 \| \| --- \| \| <.0001 \| \| 60 \| | \| 0.66741 \| \| --- \| \| <.0001 \| \| 58 \| | \| 0.88437 \| \| --- \| \| <.0001 \| \| 61 \| | \| 0.91860 \| \| --- \| \| <.0001 \| \| 61 \| |
| \| **Pos_05_MethPercentage** \| \| --- \| \|  \| | \| 0.67394 \| \| --- \| \| <.0001 \| \| 61 \| | \| 0.84649 \| \| --- \| \| <.0001 \| \| 61 \| | \| 0.67904 \| \| --- \| \| <.0001 \| \| 61 \| | \| 0.89608 \| \| --- \| \| <.0001 \| \| 61 \| | \| 1.00000 \| \| --- \| \|  \| \| 61 \| | \| 0.45958 \| \| --- \| \| 0.0002 \| \| 61 \| | \| 0.37911 \| \| --- \| \| 0.0033 \| \| 58 \| | \| 0.45570 \| \| --- \| \| 0.0002 \| \| 61 \| | \| 0.74826 \| \| --- \| \| <.0001 \| \| 61 \| | \| 0.79661 \| \| --- \| \| <.0001 \| \| 47 \| | \| 0.82702 \| \| --- \| \| <.0001 \| \| 60 \| | \| 0.69664 \| \| --- \| \| <.0001 \| \| 58 \| | \| 0.82305 \| \| --- \| \| <.0001 \| \| 61 \| | \| 0.90201 \| \| --- \| \| <.0001 \| \| 61 \| |
| \| **Pos_06_MethPercentage** \| \| --- \| \|  \| | \| 0.27845 \| \| --- \| \| 0.0298 \| \| 61 \| | \| 0.48239 \| \| --- \| \| <.0001 \| \| 61 \| | \| 0.74794 \| \| --- \| \| <.0001 \| \| 61 \| | \| 0.43947 \| \| --- \| \| 0.0004 \| \| 61 \| | \| 0.45958 \| \| --- \| \| 0.0002 \| \| 61 \| | \| 1.00000 \| \| --- \| \|  \| \| 61 \| | \| 0.01168 \| \| --- \| \| 0.9306 \| \| 58 \| | \| -0.03895 \| \| --- \| \| 0.7657 \| \| 61 \| | \| 0.32713 \| \| --- \| \| 0.0101 \| \| 61 \| | \| 0.55309 \| \| --- \| \| <.0001 \| \| 47 \| | \| 0.40287 \| \| --- \| \| 0.0014 \| \| 60 \| | \| 0.82341 \| \| --- \| \| <.0001 \| \| 58 \| | \| 0.39685 \| \| --- \| \| 0.0015 \| \| 61 \| | \| 0.59887 \| \| --- \| \| <.0001 \| \| 61 \| |
| \| **Pos_07_MethPercentage** \| \| --- \| \|  \| | \| 0.61504 \| \| --- \| \| <.0001 \| \| 58 \| | \| 0.55052 \| \| --- \| \| <.0001 \| \| 58 \| | \| 0.13224 \| \| --- \| \| 0.3224 \| \| 58 \| | \| 0.45853 \| \| --- \| \| 0.0003 \| \| 58 \| | \| 0.37911 \| \| --- \| \| 0.0033 \| \| 58 \| | \| 0.01168 \| \| --- \| \| 0.9306 \| \| 58 \| | \| 1.00000 \| \| --- \| \|  \| \| 58 \| | \| 0.44354 \| \| --- \| \| 0.0005 \| \| 58 \| | \| 0.58997 \| \| --- \| \| <.0001 \| \| 58 \| | \| 0.33546 \| \| --- \| \| 0.0243 \| \| 45 \| | \| 0.60429 \| \| --- \| \| <.0001 \| \| 57 \| | \| 0.07645 \| \| --- \| \| 0.5791 \| \| 55 \| | \| 0.62653 \| \| --- \| \| <.0001 \| \| 58 \| | \| 0.57075 \| \| --- \| \| <.0001 \| \| 58 \| |
| \| **Pos_08_MethPercentage** \| \| --- \| \|  \| | \| 0.21344 \| \| --- \| \| 0.0986 \| \| 61 \| | \| 0.33512 \| \| --- \| \| 0.0083 \| \| 61 \| | \| -0.01410 \| \| --- \| \| 0.9141 \| \| 61 \| | \| 0.38365 \| \| --- \| \| 0.0023 \| \| 61 \| | \| 0.45570 \| \| --- \| \| 0.0002 \| \| 61 \| | \| -0.03895 \| \| --- \| \| 0.7657 \| \| 61 \| | \| 0.44354 \| \| --- \| \| 0.0005 \| \| 58 \| | \| 1.00000 \| \| --- \| \|  \| \| 61 \| | \| 0.59816 \| \| --- \| \| <.0001 \| \| 61 \| | \| 0.15533 \| \| --- \| \| 0.2972 \| \| 47 \| | \| 0.74770 \| \| --- \| \| <.0001 \| \| 60 \| | \| -0.05244 \| \| --- \| \| 0.6958 \| \| 58 \| | \| 0.50798 \| \| --- \| \| <.0001 \| \| 61 \| | \| 0.43187 \| \| --- \| \| 0.0005 \| \| 61 \| |
| \| **Pos_09_MethPercentage** \| \| --- \| \|  \| | \| 0.63784 \| \| --- \| \| <.0001 \| \| 61 \| | \| 0.75411 \| \| --- \| \| <.0001 \| \| 61 \| | \| 0.48254 \| \| --- \| \| <.0001 \| \| 61 \| | \| 0.71160 \| \| --- \| \| <.0001 \| \| 61 \| | \| 0.74826 \| \| --- \| \| <.0001 \| \| 61 \| | \| 0.32713 \| \| --- \| \| 0.0101 \| \| 61 \| | \| 0.58997 \| \| --- \| \| <.0001 \| \| 58 \| | \| 0.59816 \| \| --- \| \| <.0001 \| \| 61 \| | \| 1.00000 \| \| --- \| \|  \| \| 61 \| | \| 0.68113 \| \| --- \| \| <.0001 \| \| 47 \| | \| 0.85697 \| \| --- \| \| <.0001 \| \| 60 \| | \| 0.53048 \| \| --- \| \| <.0001 \| \| 58 \| | \| 0.82658 \| \| --- \| \| <.0001 \| \| 61 \| | \| 0.83981 \| \| --- \| \| <.0001 \| \| 61 \| |
| \| **Pos_10_MethPercentage** \| \| --- \| \|  \| | \| 0.59327 \| \| --- \| \| <.0001 \| \| 47 \| | \| 0.83083 \| \| --- \| \| <.0001 \| \| 47 \| | \| 0.66416 \| \| --- \| \| <.0001 \| \| 47 \| | \| 0.76610 \| \| --- \| \| <.0001 \| \| 47 \| | \| 0.79661 \| \| --- \| \| <.0001 \| \| 47 \| | \| 0.55309 \| \| --- \| \| <.0001 \| \| 47 \| | \| 0.33546 \| \| --- \| \| 0.0243 \| \| 45 \| | \| 0.15533 \| \| --- \| \| 0.2972 \| \| 47 \| | \| 0.68113 \| \| --- \| \| <.0001 \| \| 47 \| | \| 1.00000 \| \| --- \| \|  \| \| 47 \| | \| 0.66927 \| \| --- \| \| <.0001 \| \| 46 \| | \| 0.77919 \| \| --- \| \| <.0001 \| \| 47 \| | \| 0.77821 \| \| --- \| \| <.0001 \| \| 47 \| | \| 0.87875 \| \| --- \| \| <.0001 \| \| 47 \| |
| \| **Pos_11_MethPercentage** \| \| --- \| \|  \| | \| 0.63284 \| \| --- \| \| <.0001 \| \| 60 \| | \| 0.78069 \| \| --- \| \| <.0001 \| \| 60 \| | \| 0.50361 \| \| --- \| \| <.0001 \| \| 60 \| | \| 0.78166 \| \| --- \| \| <.0001 \| \| 60 \| | \| 0.82702 \| \| --- \| \| <.0001 \| \| 60 \| | \| 0.40287 \| \| --- \| \| 0.0014 \| \| 60 \| | \| 0.60429 \| \| --- \| \| <.0001 \| \| 57 \| | \| 0.74770 \| \| --- \| \| <.0001 \| \| 60 \| | \| 0.85697 \| \| --- \| \| <.0001 \| \| 60 \| | \| 0.66927 \| \| --- \| \| <.0001 \| \| 46 \| | \| 1.00000 \| \| --- \| \|  \| \| 60 \| | \| 0.52557 \| \| --- \| \| <.0001 \| \| 57 \| | \| 0.85807 \| \| --- \| \| <.0001 \| \| 60 \| | \| 0.87914 \| \| --- \| \| <.0001 \| \| 60 \| |
| \| **Pos_12_MethPercentage** \| \| --- \| \|  \| | \| 0.49032 \| \| --- \| \| <.0001 \| \| 58 \| | \| 0.67975 \| \| --- \| \| <.0001 \| \| 58 \| | \| 0.86915 \| \| --- \| \| <.0001 \| \| 58 \| | \| 0.66741 \| \| --- \| \| <.0001 \| \| 58 \| | \| 0.69664 \| \| --- \| \| <.0001 \| \| 58 \| | \| 0.82341 \| \| --- \| \| <.0001 \| \| 58 \| | \| 0.07645 \| \| --- \| \| 0.5791 \| \| 55 \| | \| -0.05244 \| \| --- \| \| 0.6958 \| \| 58 \| | \| 0.53048 \| \| --- \| \| <.0001 \| \| 58 \| | \| 0.77919 \| \| --- \| \| <.0001 \| \| 47 \| | \| 0.52557 \| \| --- \| \| <.0001 \| \| 57 \| | \| 1.00000 \| \| --- \| \|  \| \| 58 \| | \| 0.57028 \| \| --- \| \| <.0001 \| \| 58 \| | \| 0.78280 \| \| --- \| \| <.0001 \| \| 58 \| |
| \| **Pos_13_MethPercentage** \| \| --- \| \|  \| | \| 0.75710 \| \| --- \| \| <.0001 \| \| 61 \| | \| 0.94627 \| \| --- \| \| <.0001 \| \| 61 \| | \| 0.61381 \| \| --- \| \| <.0001 \| \| 61 \| | \| 0.88437 \| \| --- \| \| <.0001 \| \| 61 \| | \| 0.82305 \| \| --- \| \| <.0001 \| \| 61 \| | \| 0.39685 \| \| --- \| \| 0.0015 \| \| 61 \| | \| 0.62653 \| \| --- \| \| <.0001 \| \| 58 \| | \| 0.50798 \| \| --- \| \| <.0001 \| \| 61 \| | \| 0.82658 \| \| --- \| \| <.0001 \| \| 61 \| | \| 0.77821 \| \| --- \| \| <.0001 \| \| 47 \| | \| 0.85807 \| \| --- \| \| <.0001 \| \| 60 \| | \| 0.57028 \| \| --- \| \| <.0001 \| \| 58 \| | \| 1.00000 \| \| --- \| \|  \| \| 61 \| | \| 0.93239 \| \| --- \| \| <.0001 \| \| 61 \| |
| \| **MeanMethylation** \| \| --- \| \|  \| | \| 0.77951 \| \| --- \| \| <.0001 \| \| 61 \| | \| 0.93644 \| \| --- \| \| <.0001 \| \| 61 \| | \| 0.76983 \| \| --- \| \| <.0001 \| \| 61 \| | \| 0.91860 \| \| --- \| \| <.0001 \| \| 61 \| | \| 0.90201 \| \| --- \| \| <.0001 \| \| 61 \| | \| 0.59887 \| \| --- \| \| <.0001 \| \| 61 \| | \| 0.57075 \| \| --- \| \| <.0001 \| \| 58 \| | \| 0.43187 \| \| --- \| \| 0.0005 \| \| 61 \| | \| 0.83981 \| \| --- \| \| <.0001 \| \| 61 \| | \| 0.87875 \| \| --- \| \| <.0001 \| \| 47 \| | \| 0.87914 \| \| --- \| \| <.0001 \| \| 60 \| | \| 0.78280 \| \| --- \| \| <.0001 \| \| 58 \| | \| 0.93239 \| \| --- \| \| <.0001 \| \| 61 \| | \| 1.00000 \| \| --- \| \|  \| \| 61 \| |

Figure s1: Correlation of methylation levels according for each position (including the mean methylation)

**ROC analysis for all methylation positions**

In order to examine the discriminative power of each individual methylation position there was performed ROC analysis for all positions. The results are presented in table s2.

According to these results the percentage of methylation in all positions (except position 8), as well as the mean methylation level, have excellent correlation for the histological status of women harboring endometrial lesions, therefore almost all methylation positions can serve as predictors of the woman histological status.

| **ROC Association Statistics** | | | | | | | |
| --- | --- | --- | --- | --- | --- | --- | --- |
| **ROC Model** | **Mann-Whitney** | | | | **Somers' D (Gini)** | **Gamma** | **Tau-a** |
|  | **Area** | **Standard Error** | **95% Wald Confidence Limits** | |  |  |  |
| **Pos_01_MethPercentage** | 0.8854 | 0.0607 | 0.7665 | 1.0000 | 0.7708 | 0.7708 | 0.2347 |
| **Pos_02_MethPercentage** | 0.8993 | 0.0666 | 0.7688 | 1.0000 | 0.7986 | 0.7986 | 0.2431 |
| **Pos_03_MethPercentage** | 0.8785 | 0.0929 | 0.6965 | 1.0000 | 0.7569 | 0.7569 | 0.2304 |
| **Pos_04_MethPercentage** | 0.9375 | 0.0634 | 0.8133 | 1.0000 | 0.8750 | 0.8750 | 0.2664 |
| **Pos_05_MethPercentage** | 0.8542 | 0.0943 | 0.6694 | 1.0000 | 0.7083 | 0.7083 | 0.2156 |
| **Pos_06_MethPercentage** | 0.7813 | 0.0876 | 0.6095 | 0.9530 | 0.5625 | 0.5625 | 0.1712 |
| **Pos_07_MethPercentage** | 0.8472 | 0.0603 | 0.7291 | 0.9654 | 0.6944 | 0.6944 | 0.2114 |
| **Pos_08_MethPercentage** | 0.7361 | 0.1249 | 0.4913 | 0.9809 | 0.4722 | 0.4722 | 0.1438 |
| **Pos_09_MethPercentage** | 0.8472 | 0.0968 | 0.6574 | 1.0000 | 0.6944 | 0.6944 | 0.2114 |
| **Pos_10_MethPercentage** | 0.8698 | 0.0817 | 0.7097 | 1.0000 | 0.7396 | 0.8099 | 0.2252 |
| **Pos_11_MethPercentage** | 0.8160 | 0.1078 | 0.6046 | 1.0000 | 0.6319 | 0.6319 | 0.1924 |
| **Pos_12_MethPercentage** | 0.8472 | 0.0854 | 0.6799 | 1.0000 | 0.6944 | 0.6944 | 0.2114 |
| **Pos_13_MethPercentage** | 0.8924 | 0.0704 | 0.7544 | 1.0000 | 0.7847 | 0.7847 | 0.2389 |
| **MeanMethylation** | 0.9653 | 0.0360 | 0.8947 | 1.0000 | 0.9306 | 0.9306 | 0.2833 |

Table s2: Statistical characteristics of ROC curves for the methylation percentage of all positions in relation to the cytological outcome using as cut of Atypical+

**Overall accuracy for varying threshold values**

The overall accuracy for the training set, test set and the complete data set using varying threshold values of the mean methylation are presented in table s2.


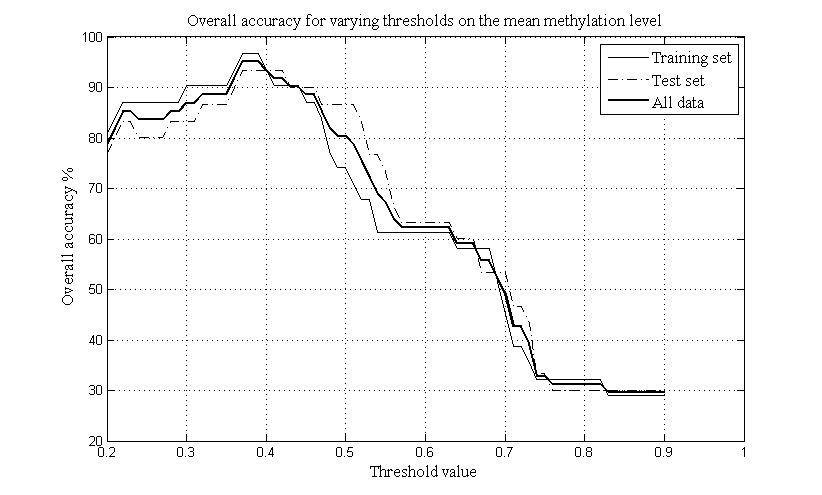


Figure s2: Threshold value vs. overall accuracy
